# Studies on the Functional Properties of Titanium Dioxide Nanoparticles Distributed in Silyl–Alkyl Bridged Polyaniline-Based Nanofluids

**DOI:** 10.3390/nano13162332

**Published:** 2023-08-14

**Authors:** Chandravadhana Arumugam, Nandakumar Velu, Padmanaban Radhakrishnan, Vellaisamy A. L. Roy, Gopalan Anantha-Iyengar, Dong-Eun Lee, Venkatramanan Kannan

**Affiliations:** 1 Department of Physics, Sri Chandrasekharendra Saraswathi Viswa Mahavidyalaya, Enathur, Kanchipuram 631561, India; chandu.vadhana@gmail.com (C.A.); vnandakumar1981@gmail.com (N.V.); padhu.mphil@gmail.com (P.R.); 2 Department of Physics, S.A. Engineering College), Chennai 600077, India; 3 Department of Physics, Maharani’s Science College for Women, Mysuru 570005, India; 4 School of Science and Technology, Hong Kong Metropolitan University, Hong Kong, China; vroy@hkmu.edu.hk; 5 Intelligent Construction Automation Center, Kyungpook National University, Daegu 41566, Republic of Korea; dolee@knu.ac.kr; 6 School of Architecture, Civil, Environment and Energy, Kyungpook National University, 1370, Sangyeok-dong, Buk-gu, Daegu 702701, Republic of Korea

**Keywords:** titanium dioxide, polyaniline, silyl–alkyl groups, nanofluids, thermal conductivity, stability, thermophysical properties

## Abstract

In the present work, a new kind of nanocomposite (NC)-based solid component was prepared for formulating nanofluids (NFs). The NC comprised metal oxide (titanium dioxide, TiO_2_) dispersed in a conducting polymer with polyaniline (PANI) and chemically linked silyl–alkyl units in it (PSA) that were designated as T-PSA NC. The NFs with ethylene glycol (EG) as a base fluid were prepared with T-PSA NCs with various compositions of TiO_2_ and PSA as well for various concentrations of T-PSA NCs. The scanning electron microscopic evaluation of the NC revealed that PSA deposition on TiO_2_ nanoparticles (NPs) decreased particle agglomeration. The PSA coating on the TiO_2_ NPs did not influence the crystalline structure of the TiO_2_ NPs, according to the X-ray diffraction patterns. The thermophysical characterization and molecular interaction features of the NFs at 303 K including a novel inorganic–organic T-PSA NC, were detailed. Furthermore, the stability of the T-PSA NC-based NFs was investigated experimentally using the zeta potential, and the particle size distribution change was analyzed using the dynamic light scattering (DLS) method. The T-PSA NCs had particle sizes that were significantly bigger than pristine PSA and pure TiO_2_. Most of the preparation conditions used to produce the T-PSA NCs resulted in moderately stable suspensions in EG. The results revealed that the ultrasonic velocity increased with the increase in the concentration of T-PSA NC mass % in the NFs, the refractive index and thermal conductivity increased with the increase in the concentration, and the surface tension exhibited a linear change when the ratio of mass % concentration of the T-PSA NCs increased. The combined presence of components that synergistically contribute to the electro, thermal, optical, and rheological properties is expected to attract advanced applications for NFs.

## 1. Introduction

For the last few decades, scientists have focused their attention on the development of heat transfer enhancement methodologies/approaches due to their importance in both conventional and emerging technologies [1]. It has been well demonstrated that among the three methods (active, passive, and composite) that have been successfully developed for enhancing the heat transfer process, the composite approach composed of NPs (NPs) dispersed in a base fluid (termed as nanofluids, NFs) exhibits superior performance due to the intriguing thermal behavior and related properties of these nanofluids [2]. Various researchers have revealed that the presence of nano-sized particles in a base liquid leads to improved thermal characteristics [3,4]. Such NFs also increased heat transmission during pool boiling in several situations [5,6]. A few studies by Lee et al. [7], Xie et al. [8], and Das et al. [9] demonstrated the correlation between the particle size of single NPs and the thermal conductivity (TC) of NFs containing 20–60 nm spherical NPs in water and concluded that the TC of NFs decreases with their particle size. It must be noted that the high surface area and van der Waals forces of attraction between NPs generally result in agglomeration and cluster formation, leading to larger-sized clumps that can have a disadvantageous influence on the stability, physical properties, and fluid flow characteristics of NFs. While there are different kinds of NPs, like metal-, metal-oxide- and carbon-based nanomaterials, that are available for adequate thermal transfer enhancement, among them, researchers prefer those that have additional properties, like stability and adaptability to working conditions [10]. These preferences have triggered extensive investigations in order to mitigate the problems associated with the application of single-NP-based NFs. A number of methods that include ultrasonic methods [11], the addition of surfactants [12], pH modification [13], surface modification [14,15], etc., have been tried in order to improve the stability of NFs. These methods can be grouped into covalent coupling or chemical modification, physical adsorption/treatment, and electrostatic bonding [16]. In recent years, the functionalization of the surface of NPs has been recognized as a promising strategy to achieve long-term stability of NFs [17]. Plasma treatment has been applied as a physical treatment to change the surface of diamond NPs toward the enhancement of the dispersion stability of NFs in water [18]. Functionalization/modification (coating of the surface of NPs) has been attempted to reduce the surface energy, leading to NFs with an improved stability [19]. Considering the availability various kinds of NPs and the variations in the methods of surface modification, to the best of our knowledge, studies on the effects of surface modification on the properties of NFs are scarce.

TiO_2_ has several superior performance characteristics, like wider applications, availability at an industrial scale with a lesser cost, chemical stability, and excellent dispersibility in both polar and non-polar solvents, compared to other materials (metals and carbon nanomaterials). Studies on TiO_2_-nanostructure-based NFs have received special attention, especially in thermal solar collectors [20]. A few studies on TiO_2_-NP-/water-based NFs have clearly demonstrated their applicability in solar collectors [21,22]. Studies have been directed to understand the dependencies of the thermal conductivity and other physicochemical properties like viscosity, density, etc., on the size, shape, and volume fraction of NPs for TiO_2_-based NFs [23]. Studies have been performed to establish the influence of surfactant-modified titania on the stability and other thermophysical properties of NFs. The influence of the surfactant concentration and sonication on the stability of TiO_2_-based NFs has been reported [4,24]. Microstructural/morphological changes due to surface modification have also been shown to influence the dispersion stability of titania-based NFs [25]. Studies on surface-modified TiO_2_ NPs with amino propyl trimethoxysilane demonstrated a better dispersion stability for these NPs in organic liquids [26]. The surface modification of TiO_2_ NPs is affected by 3-isocyanatopropyltrimethoxy silane, which causes an adequate change in the zeta potential of NPs, resulting in an increased dispersibility for NPs in NFs [27,28]. TiO_2_ NPs that are surface-modified with stearic acid have been shown to extend the lifetime of oil-based NHs [29].

Conjugated polymers (CPs) possess carbon–carbon double bonds (C=C, ~610 kJ/mol), a rigid, conjugated backbone, and strong intermolecular p-p stacking interactions that cause them to have an improved phonon transport ability along the polymer chains and, hence, make them suited for use as thermal conductors [30]. Typically, the significantly stronger p-p stacking interaction between the chains in CPs (10 to 100 times stronger than the weak vdW interactions) could be the reason for the enhanced phonon transport across the chains [31]. However, traditional CPs are characterized by low thermal conductivities (~0.2 W/m·K) similar to those of non-conjugated polymers due to probable phonon scattering [32]. Among the CPs, polyaniline (PANI), with its excellent stability, chemical redox reversibility, unique doping/de-doping processes, nanostructure, and composite forming capabilities, has emerged as a prominent thermoelectric material [33]. A nearly 140% enhancement in heat transfer properties has been witnessed for PANI/water NFs due to the unique morphology (nanofibers) and crystalline nature of PANI [34]. In another report, about 10.5% and 69.6% heat transfer improvements were observed for 0.1% and 0.5% inclusion of PANI in NFs [35]. The mass loading-dependent heat transfer effects were noted in another report [36]. Enhanced thermal conductivity behavior was witnessed in a few PANI nanofiber-based NFs [35,37]. Considering the low thermal conductivity of pristine PANI, a variety of PANI-based composites have been extensively investigated to improve the heat transfer features [38]. Various kinds of inorganic materials have been selected as thermoelectric property enhancers to incorporate with PANI, which includes metal oxides [39]. The 12% to 38% enhancement in heat transfer has been reported when PANI was coupled with CuO in a nanocomposite form [40]. In another report, the NF prepared with 10 wt.% CuO-PANI nanocomposites exhibited 31.34% heat transfer enhancement [41]. The thermo-optical response of poly (aniline-co-ortho phenylenediamine)@TiO_2_ composite was reported [42]. Various strategies have been evolved to enhance/optimize the thermoelectric performance of PANI-based composites, which include doping the PANI structure [43], molecular self-assembly [44], and inorganic nanoparticle incorporation [45]. A comprehensive literature search informed us that the PANI-based NFs have the potential to significantly improve the thermophysical properties of NFs, and the judicious selection of inclusion of components in the PANI composite is very much essential to enabling improved heat transfer performance. However, the literature search revealed that studies on the fabrication of NFs based on chemically modified PANI and TiO_2_-based NCs are limited.

**Scheme 1:**
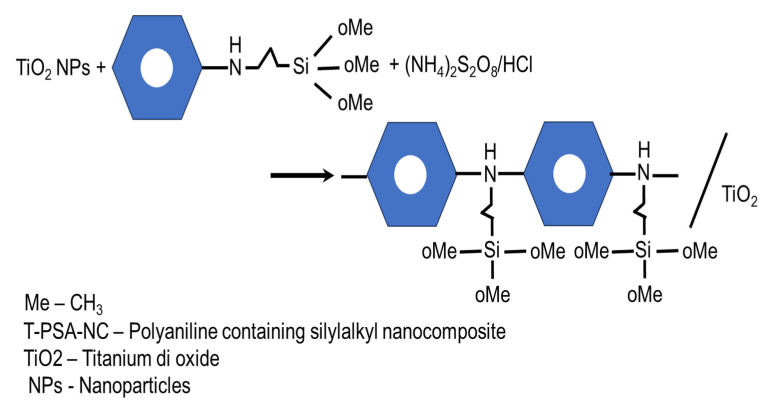
Preparation of T-PPSA NC from TIO_2_ NPs and NPAPTMS.

In the present work, the formulation and thermophysical characterization of the new NFs comprising a novel inorganic–organic nanocomposite, TiO_2_- PANI chains bridged with alkyl silyl units (designated as T-PSA NC, Scheme 1), has been reported for the first time. The literature informed us that few studies clearly demonstrated that NFs that were formulated with TiO_2_-SiO_2_ in water:ethylene glycol (EG) enhanced the heat transfer characteristics [46,47]. The TiO_2_-SiO_2_ materials used in the abovementioned studies were physical mixtures. In this work, we report the NF’s formulation based on the NC prepared with the dispersion of TiO_2_ into a silyl–alkyl containing PANI (PSA) matrix. Importantly, a simple two-step method was employed for the formulation NF that includes (i) the one-step preparation of T-PSA NC and (ii) the dispersion of T-PSA NC in the base fluid, ethylene glycol (EG). The present method offers an easy and rapid procedure for the formulation of a novel NF containing CP, silyl–alkyl containing PANI, and TiO_2_, with greater control over the composition of the NCs. The thermophysical properties of conductivity of T-PSA NC-based NFs were evaluated at different compositions and NC mass fractions (0.1–2.0%) in T-PSA NC. The results of various thermophysical properties were examined, analyzed and discussed with respect to the effects of mass fraction (%) and concentrations of T-PSA in the NFs. 

## 2. Methodology

### 2.1. Materials

Titanium (IV) oxide NPs (TiO_2_ NPs), anatase (less than 25 nm size), and N-Phenyl-3-aminopropyltrimethoxysilane (NPAPTMS) (molecular formula, C12H21NO3Si) were purchased from Sigma Aldrich. Ammonium peroxodisulfate (APDS) (99.9%, Merck, India), concentrated hydrochloric acid (HCl) (Sisco Research Laboratories Pvt. Ltd., Mumbai, India) and EG were purchased and used without further purification. 

### 2.2. Preparation of T-PSA NC-Based NFs

#### 2.2.1. Step 1: Preparation of T-PSA NCs

In a typical preparation of T-PSA NC, polymerization of 1.09 mL of NPAPTMS was carried out at 5 °C in 40 mL of 0.1M HCl in the presence of finely distributed and calculated quantity of TiO_2_ (0.5 g) by stirring prior to polymerization, The polymerization of NPAPTMS was carried out for 1 h with the addition of ammonium persulfate solution (1.14 g in 10 mL) and continuous stirring, keeping the temperature at 5 °C (Scheme 1). After that, the green mass was washed with 0.1 M HCl and filtered repeatedly for a few times until the filtrate was colorless. The green mass (designated as T-PSA NC-1) was dried in oven at 80 °C overnight. The other T-PSA NCs were prepared with varying NPAPTMS, TiO_2_, and APDS conditions and designated as described in Table 1. For comparative purposes, PSA was prepared alone in the experimental condition, similar to the preparation of T-PSA NC-1, but in the absence of TiO_2_ NPs. 

#### 2.2.2. Step 2: Formulation of T-PSA NC-Based NFs

**Scheme 2:**
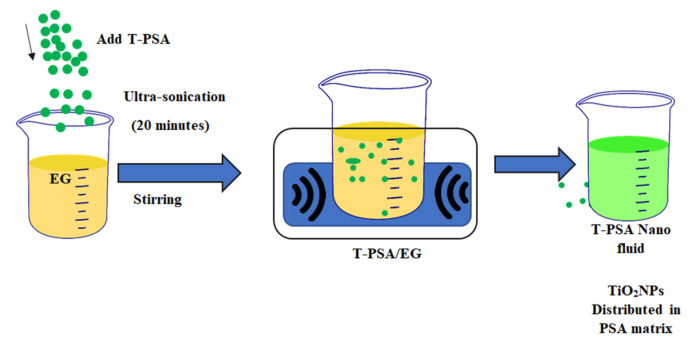
Formulation of TiO_2_ NPs distributed in the matrix of polyaniline containing silyl–alkyl-unit-based nanocomposite (T-PSA-NC) and ethylene glycol (EG) base fluid.

The preparation of T-PSA NC-based NFs as stable suspensions with adequate stability is given importance. The typical conditions selected for the preparation of T-PSA NC-based NF are described. The typical procedure involved the dispersion of 0.1 g of the NC in 100 mL of EG under a magnetic stirrer, initially for 20 min, and subsequently with ultra-sonication for 20 min (Scheme 2). Considering the number of samples that need to be analyzed for further characterization and property measurements, we selected the above fixed condition for the preparation of NFs of all the samples. Hence, detailed studies on capturing images and stability characterization have not been performed. Please note that the stability data of NF collected in this work and detailed in the discussion part with T-PSA NCs showed moderate stability in the used conditions. Scheme 2 depicts a pictorial presentation of the NF preparation procedure used in this work.

**Scheme 3:**
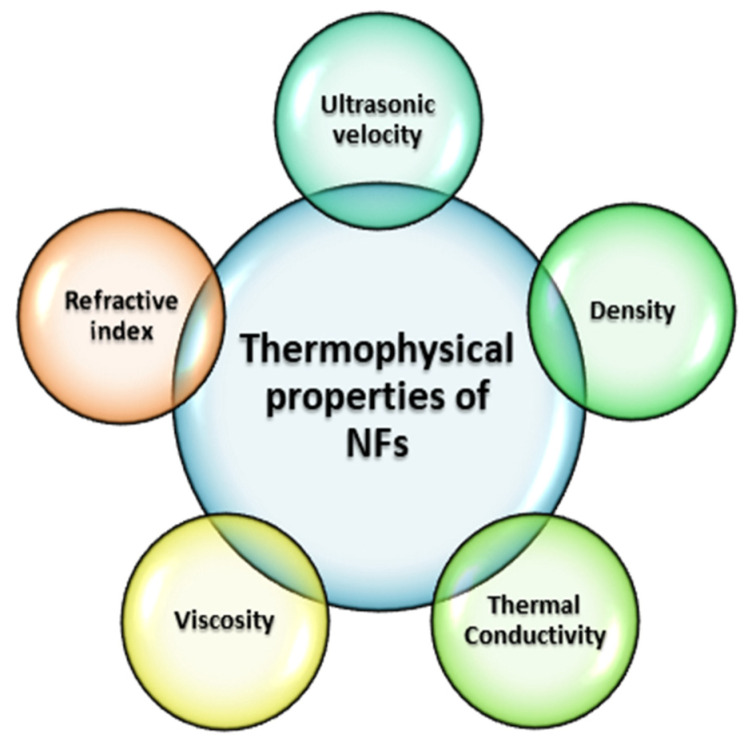
Thermophysical properties of T-PSA-NCs studied in this work.

Scheme 3 presents the various thermophysical properties that were deduced for the T-PSA-NC.

### 2.3. Characterization of Properties of T-PSA NC-Based NFs

The ultrasonic velocity of the NFs was evaluated at the temperature of 303 K using the MITTAL single-frequency ultrasonic interferometer (2 MHz, model F-81). The NFs’ viscosity at 303 K was determined using a digital viscometer (BROOKFIELD brand, Haverhill, MA, USA). The density of the NFs was calculated using a pyknometric approach. The mass of the liquid was determined using an electronic single-pan balance (made by K-ROY, Kolkatta, India) with an accuracy of 0.001 g. The refractive index was determined with an accuracy of 0.001 using Mittal Abbe refractometer (New Delhi, India). A thermostat with a temperature measuring accuracy of ±0.05 K was used to circulate water at 303 K during the measurement of the properties of NFs using viscometer, interferometer, and refractometer. The zeta potential values were measured using the Zetasizer (Litesizer 500, Malvern Pananalytical Ltd., Malvern, UK) instrument. The particle size, zeta potential, and molecular weight of the solution were determined by integrating dynamic light scattering, laser Doppler micro electrophoresis, and static light scattering.

## 3. Results and Discussion

### 3.1. Morphology and Microstructure of T-PSA NC

**Figure 1:**
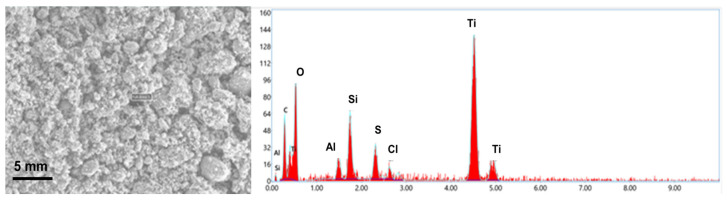
SEM image and EDAX of a typical T-PSA NC.

An SEM image of T-PSA NC shows the presence of highly dispersed agglomerated particles with an interlocking arrangement between the particles (Figure 1). It is therefore considered that most of the TiO_2_ NPs were coated with PSA during the polymerization step. The EDX spectrum (Figure 1) indicates the presence of three major elements: Ti, Si, and O. The N K-alpha (392 eV) was expected to overlap with Ti and hence could not be found separately. The XRD analysis of the typical T-PSA-NC (not shown) exhibits a series of diffraction peaks that correspond to the crystal planes of (001), (004), (020), (015), (024), and (204), representing the tetragonal anatase phase of TiO_2_ (JCPDS file No: 86-1157). Broad reflections around 20° and 25.90° indicated the amorphous form of PANI fragments in PSA [48]. 

### 3.2. Importance of EG as Base Fluid for T-PSA NC

Many base fluids, such as aqueous and organic liquids (EG and oils, etc.) have been utilized to formulate NFs toward the enhancement of the thermophysical properties of NFs. The TC enhancement depends on the NPs base fluid compositions [49]. EG is a commercially available solvent known to be used in automobiles as an engine coolant. In the present work, EG was chosen as the base fluid for the preparation of T-PSA NC-based NFs. Importantly, the literature reveals the significance of EG as a base fluid in metal oxide and PANI-based NFs, as well as in modulating the morphology and particle size distribution of various PANI nanostructures. The results on oxide-based NFs (A1_2_O_3_ in EG and CuO in EG) revealed that the TC of the CuO in the EG system can be enhanced by more than 20% as compared to the base fluid [50]. The use of a 60:40% water–EG mixture as a base fluid for nanodiamond–Fe_3_O_4_ hybrid-based NF was found to exhibit thermal heat transfer enhancements of 5.03% and 12.79% and viscosity enhancements of 108% and 50.84% at temperatures of 20 °C and 60 °C, respectively [51]. EG plays a key role in the formation of PANI particles with controlled distribution and defined morphology [52]. PANI nanorods with good dispersibility in solvents (water and alcohol) were prepared by the chemical oxidative method in an EG medium [53]. An ink composed of PANI in EG (along with other solvents) with an adequate viscosity was developed for ink jet printing [54]. The mixture of water and EG was used for the preparation of polypyrrole/TiO_2_/PANI ternary nanotube hybrids [55]. The uniformly distributed graphene/PANI hybrid material was prepared in an EG medium [56]. The role of EG was demonstrated for the preparation of PANI nanospheres with unique morphology [57].

### 3.3. Thermophysical Properties of T-PSA NC in NF

#### 3.3.1. Viscosity

Table 2 presents the viscosity of the T-PSA NCs: EG NFs. The viscosity of T-PSA NCs is much lower than the viscosity of pure EG [58] and also shows dependence on the composition of the TiO_2_ and NPAPTMS used in the preparation of NC (Table 1). The T-PSA NCs prepared with increasing NPAPTMS amounts and a fixed TiO_2_ amount, namely T-PSA NC1, T-PSA NC2, and T-PSA NC3, have exhibited significantly lower viscosity compared to pure EG and showed dependence on the compositions of the components in T-PSA NCs. Similarly, the dependence of viscosity on T-PSA NCs including NFs informs a non-Newtonian behavior for the NFs. Similarly, T-PSA NC4 and T-PSA NC5, prepared with different TiO_2_ amounts (0.3 g and 0.8 g) but with a constant amount of NPAPTMS, showed much lower viscosities as compared to the base fluid. Typically, T-PSA NC4-based NF prepared with the NC containing 0.8 g of TiO_2_ (the highest amount amongst the samples) (Table 1) exhibited the lowest viscosity (Table 2). It is therefore concluded that the decrease in viscosity was synergistically influenced by the amount of TiO_2_ and NPAPTMS used in the preparation of the NC. Generally, viscosity is interpreted in terms of fluid resistance to shear stress. When the solid particles offer increasing stress, more force is required to move the fluid. In this work, it is presumed that the friction force between EG and the solid surface of T-PSA is dependent on the composition of T-PSA NC. It must be noted that a PSA matrix with -NH and siloxy (Si-O-) groups can have intermolecular hydrogen bonding interactions with EG, and the extent of the bonding interaction can depend on the proportion of the -NH groups in the NCs. Although the interactions of metal oxides and aqueous base fluids are not clearly understood, few studies have explained them in terms of hydrogen bonding interactions. The attraction of EG molecules to the surface of carbon nanotubes has been attributed in terms of hydrogen bonding interactions [59], which in turn cause good dispersion in the NFs. The effects of hydrogen bonding on viscosity and dispersions and the roles of EG are presented for Fe_2_O_3_-based NFs [60]. Considering the fact that T-PSA NCs comprise TiO_2_, silica-like structures, and PANI, we intend to explain the results on the non-Newtonian behavior of NFs in terms of the components of NCs in NFs. While NFs containing SiO_2_ NPs only revealed Newtonian behavior, the behavior of NFs with both SiO_2_ and TiO_2_ NPs is non-Newtonian [61]. In another study, the rheological behavior of SiO_2_-MWCNTs/EG NF was reported to be non-Newtonian over a wider mass fraction of the solid particles [62].

**Figure 2:**
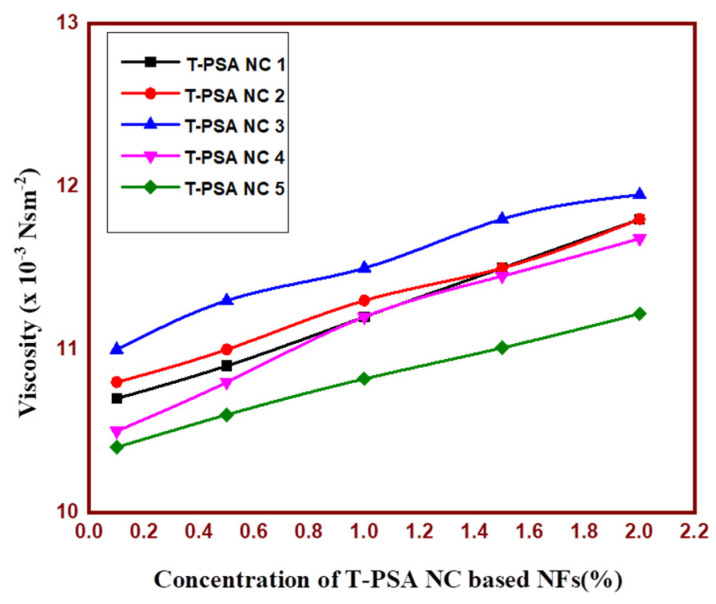
Variation of viscosity against various concentrations of T-PSA NC-based NFs.

Figure 2 presents the viscosity changes with a concentration of T-PSA NCs in the NFs. A linear variation with an increased concentration of T-PSA NCs in the NFs was witnessed. To note, T-PSA NC-5 (with the lowest amount of TiO_2_ in the preparation condition of NC) shows the lowest viscosity in the entire concentration range (Figure 2). And T-PSA NC-3 (prepared with the highest amount of NPAPTMS) shows the highest viscosity in the entire concentration range (Figure 2). It must be noted that the viscosity measurement was performed independently for various concentrations of individual NCs. The particle sizes of individual NC are expected to be nearly the same in the entire concentration. However, increasing the concentration of an NC is expected to increase the number of particles. The changes in viscosity are therefore attributed to a higher suspension concentration (number of suspended NPs) in the base fluid, which causes higher internal viscous shear stress [63]. It is imperative that the properties of NFs are affected by a number of factors, including the pH, particle aggregation, shear rate, particle shape, particle size, volume percentage of nanomaterials, and surfactants utilized [64,65]. There are contradictory discussions on the influence of viscosity on changes in particle sizes. Variations in the sizes of NPs have been reported to influence the viscosities of NFs differently. In one of the reports, an increase in the viscosity of NF and an increase in the particle size were noted [66]. On the contrary, in another report, a decrease in the viscosity with an increase in the particle size was witnessed [67]. The reason for decrease in the viscosity was attributed to the greater resistance at the nanoparticle/fluid interface. The heat transfer performance and pressure drop characteristics of ZnO/water-based NFs were reported [68]. The heat transfer enhancements of 10.6% and 13.2% were noticed for the volume concentration of NPs 0.75% and 1.5%, respectively, with the augmentation in the friction factors. The viscosity for ZnO/water-based NFs was noticed to be 8.52% at 0.012 wt.%, 12.71% at 0.024 wt.%, 16.58% at 0.036 wt.%, and 20.31% at 0.048 wt.% mass concentrations of NPs as compared to the base fluid at 40 °C [68]. The viscosity increase with the concentration of NPs was explained in terms of the increase in the interfacial forces between the adjacent layers. In the present work, the T-PSA-NC5 prepared with the lowest TiO_2_ in the preparation of NC showed distinctly lower viscosities at higher concentration ranges (Table 2). Knowing the trend of T-PSA NC-3 (highest) and T-PSA NC5 (lowest) on viscosity (Figure 2), it is presumed that particle sizes as well as the coating layer amount may play a predominant role in the viscosity values.

#### 3.3.2. Density

**Figure 3:**
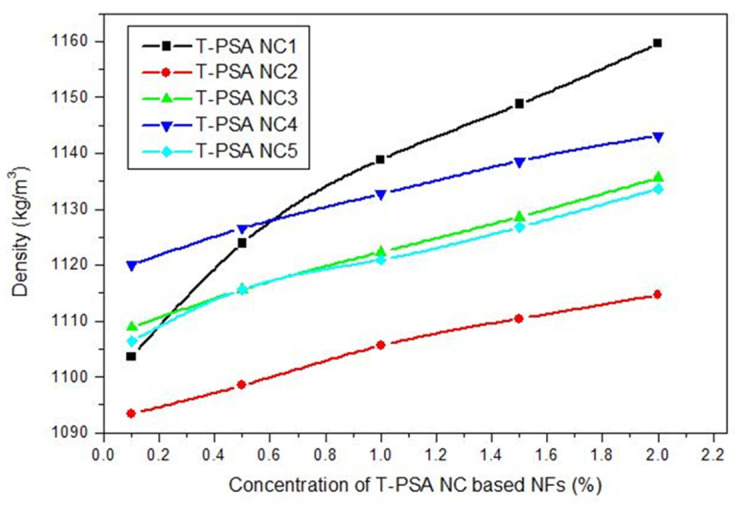
Variation of density against various concentrations of T-PSA NC-based NFs.

The density of NF is considered the most important property of a fluid because it can influence parameters such as the Reynolds number, friction factor, pump loss, etc. In this work, the density of PSA was similar to T-PSA NC-5, prepared with a small amount of TiO_2_ (Table 1 and Table 2). Interestingly, T-PSA NCs prepared with an increased amount of NPAPTMS and with a fixed amount of TiO_2_ (T-PSA NC-1, T-PSA NC-12, and T-PSA NC-3) showed a non-linear variation in density (Table 2). While a comparison of density of T-PSA NC-5, T-PSA NC-1, and T-PSA NC-4, prepared with an increasing amount of TiO_2_ but with a similar amount of NPAPTMS, showed an increasing trend in density. Considering the results, it can be concluded that density variation is strongly dependent on the TiO_2_ amount. Additionally, the polymerization of NPAPTMS can result in different extents of cross-linking in the PSA, which can cause different cross-linking densities in T-PSA NC that can synergistically influence the density. In general, density variations of mono NFs could be correlated with the concentration at a constant temperature (298 K) using theoretical formulas [69,70]. However, studies on such correlations are limited for defined composites/hybrids [71]. The prediction of the density of rGO-Fe_3_O_4_-TiO_2_ ternary hybrid/EG-based NF with the correlation over the concentration of NPs has been detailed [72]. It also must be noted that physical properties such as Gibbs free energy, internal pressure, molar volume, free volume, adiabatic compressibility, and other molecular interaction characteristics can influence the density of NFs [73,74,75]. Notably, the results from a few studies on TiO_2_/EG NFs that focused on thermal or rheological properties are available [76,77,78,79]. Studies on the TiO_2_–ZnO hybrid in the water–EG mixture with different volume concentrations up to 0.1–2.2% revealed the roles of concentration, composition, and temperature on the rheological properties of NFs [80]. In the present work, the density of T-PSA NC-based NF increased with the concentration of NCs (Figure 3). The density of T-PSA NC-5 prepared with a lower concentration of NPAPTMS has the lowest density for all the concentrations (Figure 3). Hence, it is presumed that PSA formed from NPAPTMS contributes to the density changes. On the other hand, T-PSA NC-5 showed the highest density values beyond the concentration of 1% mass of T-PSA NC in the NFs. This result corroborates the non-linear variation of density with the composition of T-PSA NC.

#### 3.3.3. Ultrasonic Velocity (USV) Measurements

USV is considered an important parameter to evaluate the intraparticle and intermolecular interactions (liquid–particle and particle–particle interactions) in the NFs with simple or composite suspended particles in the base fluid [81,82,83,84,85]. The USVs of PSA and T-PSA NC1 are closer to each other, signifying the fact that NCs prepared with lower amounts of NPAPTMS in the NC preparation condition did not change the USV. On the other hand, T-PSA NC 2 to T-PSA NC 5, prepared with different amounts of TiO_2_ and NPAPTMS in the preparation conditions, showed an increased USV compared to the base fluid. The increase in USV from T-PSA NC2 to T-PSA NC -5 is non-linear with either TiO_2_ or NPAPTMS (Table 2). It is presumed that EG can form hydrogen-bonded complexes with groups in the T-PSA NC [86]. The USV is the highest for the T-PSA NC3 NFs and the lowest for the T-PSA NC 2 (excepting T-PSA NC 1). Ultrasonic attenuation and ultrasonic velocity were studied for a polymer colloidal solution with dispersed nanoparticles and interpreted in terms of the molecular interaction between polymer and NPs [87].

**Figure 4:**
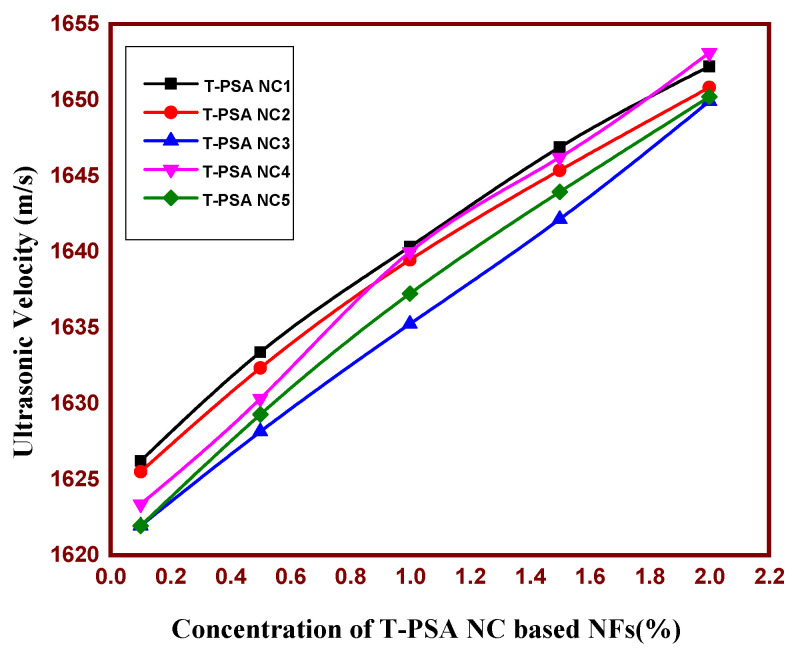
Variation of ultrasonic velocity against various concentrations of T-PSA NC-based NFs.

Figure 4 demonstrates that the USV increases with increases in the concentration of T-PSA NC in the NFs. The increase in USV for NF can be attributed to the possible interaction that arises due to effects arising from PSA on the TiO_2_ surface and the possible hydrogen bonding interaction between particles and base fluid molecules. Hence, there might be particle–fluid interactions, which may favor an increase in the velocity values [86]. 

#### 3.3.4. Refractive Index

The refractive index (n), an important optical parameter that indicates the electronic polarizability of the ions in a material, is used to decide the material for the fabrication of optoelectronic devices [88]. In the case of NFs, n represents the ratio of the speed of light in a vacuum to the speed of light in a medium. The wavelength, shape of the particles, and chemical composition of the NPs can influence n. In NPs, the refractive index is analogous to the optical force generated by an electromagnetic field. The absorption coefficient of nanoliquids can be easily calculated from the transmission spectrum if the refractive index of the base liquid (i.e., the solution without NPs) is known. It is difficult to determine the absorption coefficient merely from the transmission spectrum if the base liquid’s refractive index is unknown [89]. On perusal of Table 2, the n of T-PSA NC1 is similar to T-PSA, which is slightly higher than PANI (1.41) [90]. The n values of other T-PSA NC2 to T-PSA NC5 exhibit non-linear changes with NPAPTMS and TiO_2_. 

In general, polymers are known to possess a small, adjustable range of n values (1.3–1.7) [91]. On the contrary, inorganic/organic hybrid materials or composites can combine the lightweight features of the polymeric component with the high refractive index and UV shielding ability of the inorganic nanomaterials [92,93]. TiO_2_ nanocomposites have been proven to have a high refractive index and high transparency due to a high refractive index (n = 2.45 and 2.7 for anatase and rutile phases, respectively) and a very low absorption coefficient in the visible range for TiO_2_ NPs [94,95]. 

The higher n value for T-PSA NC1 than PANI (the type of PSA) is definitely due to the synergistic interaction between TiO_2_ NPs and PSA, as reported for the polymer nanocomposites [90]. Keeping in mind that n and bandgap energy of a material are known to have opposite trends, the higher n values for T-PSA NCs indicate a possible decrease in the optical bandgap of the NCs. From the n analysis, we can infer that the as-prepared T-PSA NC-based NF can find usefulness in fabricating optoelectronic devices. 

**Figure 5:**
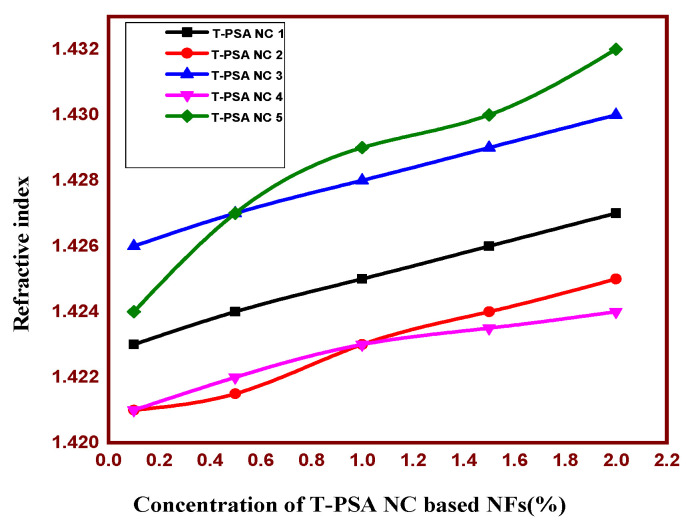
Variation of refractive index against various concentrations of T-PSA NC-based NFs.

Figure 5 presents the changes in the RI values with increasing concentrations of T-PSA NC. Figure 5 depicts the linear increase in n with an increase in the concentration of T-PSA NCs. In general, it can be observed that the n value increases as the concentration increases. The observation that T-PSA NC 5 shows the highest n at concertation higher than 0, 8% mass of NC, informs us that the PSA layer formed over TiO_2_ for NC having a lower amount of NPAPTMS contributes mainly to the reason for the increase in n.

#### 3.3.5. Thermal Conductivity

The TC of NFs is considered an important thermal property, and it strongly correlates with the heat transfer performance and thermal efficiency of the concerned devices/systems [96]. The TC of NFs can be measured using various techniques, including steady-state, transient, and thermal comparator techniques [97]. Factors such as characteristics of NPs (size, shape, concentration, etc.), temperature, stability, nature of the base fluid and measurement methods [60,98] can influence TC. While reports on the TC of metal oxide/EG-based NFs are widely available [99,100], few studies have investigated TiO_2_/EG-based NFs. [101,102,103]. Studies on the TC of a few of the PANI-based NC are available [35,40]. While Cu-PANI composite-based NFs exhibited a TC improvement of 107–159% at temperatures ranging from 10 °C to 90 °C [104], nearly 12% to 38% TC improvement was witnessed when PANI was coupled with CuO in a nanocomposite form [40]. The present study reports the TC of a different kind of silica functionalized PANI-TiO_2_ NC-based NF in EG for the first time. The TC of T-PSA NC1 to T-PSA NC5 differs from the parent PSA and shows dependence (Table 2) on the amount of TiO_2_ and NPAPTMS used for the preparation of the NCs (Table 1). The T-PSA NC2, T-PSA NC1, and -T-PSA NC3 were prepared with fixed amounts of TiO_2_ (0.5 g) and increasing amounts of NPAPTMS (Table 1). The TC of -PSA NC2, T-PSA NC1, and -T-PSA NC3 showed an increasing trend in TC with increasing amounts of NPAPTMS used in the preparation of NCs. The samples T-PSA NC5, T-PSA NC1, and -T-PSA NC4 were prepared with increasing amounts of TiO_2_ while having a fixed amount of NPAPTMS (Table 1). The T-PSA NC4 showed the largest TC value (Table 2). It is expected that increases in the amount of TiO_2_ can result in more TiO_2_ particles for coating PSA on the surface of TiO_2_. However, there can be variations in the thickness of the PSA coating on the TiO_2_ particle surface amongst -PSA NC5, T-PSA NC1, and -T-PSA NC4. Hence, the non-linear dependence of TC within -PSA NC5, T-PSA NC1, and -T-PSA NC4 is expected to be the consequence of synergistic effects of the number of particles and particle sizes. TC enhancement has already been explained with mechanisms involving liquid layering around the NP surface, ballistic phonon transport, Brownian motion-driven micro convection, and nanoparticle aggregation [105,106,107,108,109]. In this work, we propose to explain the non-linear dependence of TC in terms of nanolayer effects. The ordered solid–liquid interface, caused by the strong particle–fluid force of interaction and nanolayer formation, was considered and interpreted. It is inferred that the TC influence of the nanolayer-coated particle is much higher than that of the bulk base fluid and the pristine nanoparticles [105]. The nanolayer between T-PSA NC and EG can function as the thermal bridge between them. The concentration of T-PSA NC5 in the EG as well as the composition of T-PSA NC5 cause TC enhancement of NFs [110]. It must also be noted that the T-PSA NC5 particle–EG interface can introduce an interfacial thermal resistance (Kapitza resistance), which can antagonize the heat transfer process and can decrease the overall TC within the system. However, the high specific surface area of NPs and nanometer thickness of nanolayer are expected to play key roles in heat transfer enhancement across the particle–fluid interface [111].

**Figure 6:**
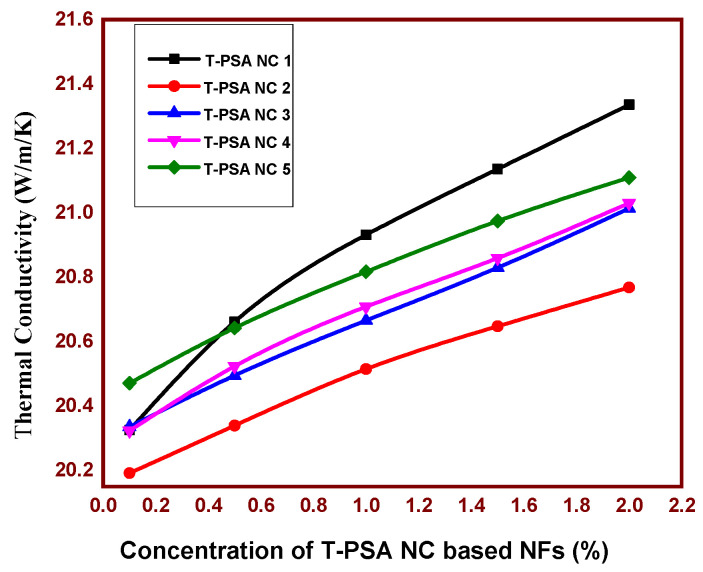
Variation of thermal conductivity against various concentrations of T-PSA. NC-based NFs.

Figure 6 shows the changes in TC of T-PSA NC for various mass % concentrations in the NFs. It is evident that TC increases with increases in T-PSA NC mass % in the NFs. It must be noted that in Figure 6, the TC of any of the NCs (from PSA N1 to T-PSA NC5) is compared with an increasing amount of NC, keeping in mind that the individual NCs are expected to have almost similar particle sizes. Hence, the observed increasing trend in TC with increasing amounts of NC is expected to be caused by the increased number of particles. Typically, the TC of T-PSA NC1 was found to be the highest amongst all of the NCs (beyond 0.5% mass in the NFs), signifying the role of PSA in heat transferability. A unique metal/metal-oxide/carbon-based NC included with TiO_2_ exhibits a TC enhancement of 66–83%, even at very low mass concentrations of TiO_2_ [112]. The enhancement in thermal conductivity has been explained in terms of clustering and aggregation-induced intermolecular forces [111,113]. The literature data on the TC enhancement (%) values of TiO_2_, PANI, and silica (as PSA contains silyl framework) with reference to base fluids and the related references are presented in the Supplementary Materials (Table S1). Taking into account the thermal conductivity value for the zero case of no particles in NF as 0.254 W/m/K, the enhancement % values are calculated and presented in the Supporting Information Table S1. The abscissa in Figure 6 has a value of 0.254 W/m/K (the value of pure base fluid). On perusal of Table S1, one can infer that the presence of PSA in the T-PSA NCs significantly enhances the TC compared to the base fluid containing pristine TiO_2_. The TC enhancement (%) values for T-PSA-NCs are much higher than the reported values of simple TiO_2_-based NF (the references are given in Table S1). It is presumed that the presence of both PANI and silyl groups in PSA may contribute to the significant enhancement of thermal conductivity.

### 3.4. Molecular Interaction Properties

The acoustic investigation permits the determination of a number of molecular interactions and thermophysical properties, such as the NF’s adiabatic compressibility (β), intermolecular free length (Lf), free volume (Fv), internal pressure (Ip), and specific acoustic impedance (Z) [114]. The experimental values of the ultrasonic velocity, viscosity, and density of T-PSA NC-based NFs are used to deduce the parameters listed in Table 3. On close perusal of the values of β for T-PSA NC2 and T-PSA NC5, the value of T-PSA NC5 is lower than β and T-PSA NC2. A decline in β is often considered closed packing and lesser ion repulsion between NPs and EG and NPs (Table 3). On the other hand, an increase in β was noticed for T-PSA NC1 compared to PSA. Thus, one could notice a non-linear change in β with the compositional changes in the T-PSA NCs. The observed non-linear change in β with the composition of T-PSA NC1 informs us that the chemical groups in PSA and TiO_2_ interact significantly and can form complexes via hydrogen bonding, as discussed earlier [112]. A similar trend as noticed for β is noted for Lf with the various T-PSA-based NFs (Table 3). These observations reinforce the fact that there is a substantial interaction between EG and molecular groups (-NH- and -Si-O-, Scheme 1) in the T-PSA NC, which can augment structural organization [75]. It can be inferred that Fv follows a reverse trend of viscosity [115]. Therefore, viscosity rather than velocity determines the free volume of our system. Additionally, while Ip increases, the value of Fv shows a decreasing trend [116,117]. As can be seen in Table 3, there is a definite tendency toward a negative connection between free volume and internal pressure. Based on Table 3, it can be noticed that the trend of Z of T-PSA-NC-based NFs is inverse to β while being directly proportional to USV. The non-linear relationship in Z amongst T-PSA NCs (Table 3) informs us of the dissociative nature of the molecular interactions in the NFs [114].

Table 3 summarizes the influence of variation in the concentration of T-PSA NC in the NF on β, Lf, Fv, Fv Z, surface tension, and relaxation time. It can be observed that the β decreases with increases in the concentration of T-PSA NC in NFs. The decrease in β signifies the strength of the molecular interaction. The strong force of attraction between molecules of EG and nanocomposites indicates the decrease in β [116]. A similar trend is observed in Lf. The interaction between particles and base fluid molecules increases the intermolecular distance between the molecules, which in turn causes impedance in the propagation of ultrasonic waves. The decrease in β and Lf supports the existence of particle–fluid interaction [82].

The Fv decreases when the internal pressure increases [117,118]. The reverse trend of Fv with Ip is clearly shown in Table 2. The higher value of Z indicates that there is a significant interaction between the particle and base fluid molecules, which may affect the structural arrangement [82]. The increasing trend of surface tension suggests that as the concentration increases, more NPs gather in the solution. The NPs become close to each other with the surface molecules. As a result, a strong, cohesive force is exerted between the NPs and liquid molecules, and hence, the surface tension of the nanofluids increases when the concentration increases [119]. The reduction in surface tension of NFs is attributed to the reduction in cohesive energy at the liquid–air interface when nano-sized particles are added to the base liquid. The Brownian motion can cause dispersion of the NPs located at the liquid–air interface to result in a newer orientation with lower levels of the total free energy of the interface. Consequently, this can reduce the surface tension [119]. In the present work, from Table 2, it can be observed that the surface tension exhibits linear changes when the ratio of mass % concentration of T-PSA NCs increases. It is due to van der Waals forces between particles at the liquid/gas interface, which can lead to an increase in the surface free energy, and thus to an increase in the surface tension [120]. The decrease in relaxation time (Table 2) indicates that it may be due to the structural relaxation process [121] and hence may arise due to the rearrangement of molecules and associated co-operative processes. 

### 3.5. Particle Size Measurements

**Figure 7:**
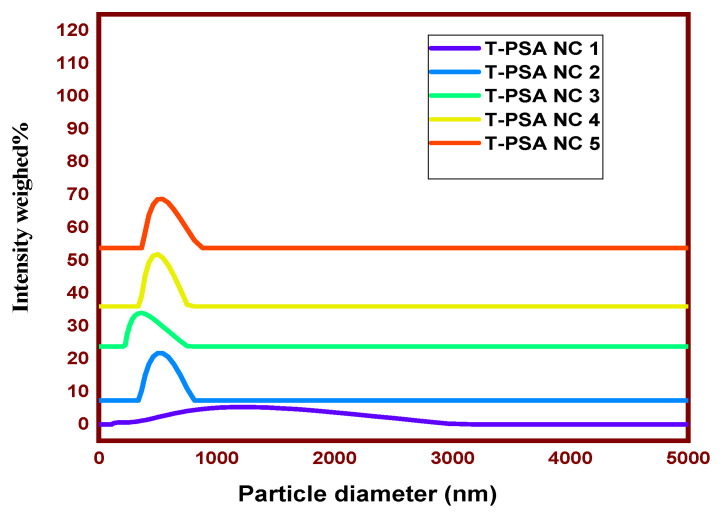
Mean diameters by DLS measurements of T-PSA NC-based NFs.

The particle size determined by DLS corresponds to (i) the light intensity scattered by the NPs and (ii) the size of particles suspended in a liquid. DL measurements of pure TiO_2_, pristine PSA, and various T-PSA NCs in the EG base fluid formulated at ambient conditions are presented in Figure 7 and Table 4. The DLS analysis reveals that T-PSA NC1 to T-PSA NC5 in EG have an average particle diameter of 1158.4 nm, 530.0 nm, 399.0 nm, 510.3 nm, and 554.2 nm, respectively. The particle sizes of T-PSA NCs are much larger than the particle sizes of pristine PSA (241.3 nm) and pure TiO_2_ (288.7 nm). It is obvious that the surface of TiO_2_ NPs is covered with a layer of PSA in the T-PSA NCs, causing an increase in particle size. The parent particle size of TiO_2_ (25 nm) was increased to 288.7 nm in EG due to the agglomeration of NPs [122]. The particle sizes of T-PSA NCs show variations amongst them due to the different extents of the PSA layer in the T-PSA NCs.

### 3.6. Zeta Potential and Stability

The zeta potential is a crucial measure for learning more about the stability of the NF. In a colloidal suspension, the electrical potential at the slip plane between a particle’s surface and dispersion liquid is referred to as the zeta potential. Taking into account Table 5, which presents the zeta potential value and the associated suspension stability, nano-suspensions can only be moderately stable at |±ξ| ≥ 30 mV [123]. It is predicted that the suspensions of pristine TiO_2_ and PSA NPs are not adequately stable in EG, and the NPs have a tendency to settle over a period of time. On the other hand, the suspensions of T-PSA NC1 to T-PSA NC4 in EG (Table 6) have far improved stability over the pristine TiO_2_ and PSA NPs. However, the suspension of T-PSA NC5 in EG is unstable (Table 6), and the reason for that could not be exactly described.

## 4. Conclusions

While conducting polymers are known to have organic-based monomeric units, the conducting polymer (PSA) used in this study has both silyl (in-organic silica frame) and alkyl (organic) groups. The solid component in the fabricated nanofluids (NFs) is expected to be a core–shell-based nanocomposite (NC) produced by dispersing titanium dioxide (T) in a polymer (PSA). The evidence for core–shell formation is evident through the comparison of scanning electron microscope images of the core and NC. Importantly, the interesting influence of the composition of core and shell components and mass concentration of NC in the newly formulated NFs demonstrate the linear dependence on various thermophysical parameters (thermal conductivity, viscosity, density, ultrasonic velocity, and refractive index) and are independently described. Notably, there is a linear change in thermal conductivity with increasing mass % concentration of T-PSA NC in the NFs and a non-linear variation with T-PSA NC composition. It is also noticed that when concentration increases, the refractive index, ultrasonic velocity, and thermal conductivity show an increasing trend. The NFs of T-PSA in ethylene glycol (EG) were moderately stable, as evident from zeta potential measurements. The changes in the particle size distribution of T-PSA N as compared to pure TiO_2_ and pristine PSA are presented. The newly produced metal oxide-conducting polymer–silica-containing NC-based NF is expected to have numerous functional (electro, rheological, thermal, and optical) features and may be used in advanced NF applications. Besides, the explanation of experimental properties like thermal conductivity requires future consideration, as such studies are scarce.

The model-based prediction of thermal conductivity for spherical particles using the Maxwell model, as reported by the majority of researchers studying the NF properties, has come to a near halt, requiring redefinition and conceptual reconsiderations. This is mainly due to the misleading conclusions and suggestions when researchers want to extend such a model to hybrids or nanocomposites. In recent years, there have been reports available on improved prediction for thermal conductivity in the form of effective medium assumptions that consider coated particles or cherry-pit (partially penetrating spheres) models (both for diffusion in composite materials and membranes and for conduction in NFs (e.g., using surfactants around the NPs)), etc. It is envisioned that for predicting the thermal conductivity of core–shell nanocomposites, as reported in this work, through models, various considerations of intrinsic parameters (like the chemical nature of the shell, thickness of the shell, shape of the shell, etc.) need to be considered.

## Figures and Tables

**Table 1:** Designation of samples.

NPAPTMS (mL)	TiO_2_ NPs (g)	APDS (g)	Designation of Samples
1.09	0.5	1.14	T-PSA-NC-1
0.59	0.5	1.14	T-PSA-NC-2
1.65	0.5	1.14	T-PSA-NC-3
1.09	0.8	1.14	T-PSA-NC-4
1.09	0.30	1.14	T-PSA-NC-5

**Table 2:** Experimental values of viscosity, density, ultrasonic velocity, and refractive index of NFs.

T-PSA NC with Varying Compositions	Viscosity (×10^−3^ Nsm^−2^)	Density (kg/m^3^)	Ultrasonic Velocity (m/s)	Refractive Index	Thermal Condutivity (W/m/K)
T-PSA NC1	10.5	1203.8	1630	1.42	21.57
T-PSA NC2	10.7	1159.5	1640	1.43	21.18
T-PSA NC3	10.6	1202.3	1660	1.42	21.95
T-PSA NC4	10.4	1218.0	1651	1.42	22.02
T-PSA NC5	10.6	1183.8	1660	1.41	21.73
PSA	10.7	1182.8	1637	1.42	21.41

**Table 3:** Various molecular interaction parameters of nanofluids at 303 K.

Name of the Sample	Concentration (%)	Adiabatic Compressibility (×10^−10^ m^2^N^−1^)	Intermolecular Free Length (×10^−11^ m)	Free Volume (×10^−9^ m^3^mol^−1^)	Internal Pressure (×10^9^ Pa)	Specific Acoustical Impedance (×10^6^ kgm^−2^s^−1^)	Surface Tension (N/m)	Relaxation Time (×10^−12^ s)
T-PSA-NC-1	0.1	3.43	3.84	3.27	2.17	1.79	43.22	4.89
	0.5	3.34	3.79	3.20	2.21	1.83	43.50	4.85
	1	3.26	3.75	3.09	2.25	1.87	43.78	4.87
	1.5	3.21	3.72	2.99	2.29	1.89	44.04	4.92
	2	3.16	3.69	2.89	2.33	1.92	44.25	4.97
T-PSA-NC-2	0.1	3.46	3.86	3.22	2.16	1.78	43.19	4.98
	0.5	3.42	3.85	3.15	2.18	1.79	43.46	5.01
	1	3.37	3.81	3.05	2.22	1.81	43.74	5.07
	1.5	3.33	3.78	2.99	2.24	1.82	43.98	5.1
	2	3.29	3.76	2.89	2.27	1.84	44.20	5.18
T-PSA-NC-3	0.1	3.43	3.84	3.13	2.21	1.79	43.04	5.03
	0.5	3.38	3.82	3.02	2.24	1.81	43.29	5.09
	1	3.33	3.79	2.96	2.26	1.83	43.58	5.11
	1.5	3.29	3.76	2.87	2.29	1.85	43.85	5.17
	2	3.23	3.73	2.83	2.32	1.87	44.16	5.15
T-PSA-NC-4	0.1	3.43	3.84	3.36	2.15	1.79	43.10	4.80
	0.5	3.37	3.81	3.24	2.19	1.82	43.38	4.86
	1	3.32	3.78	3.09	2.22	1.84	43.77	4.95
	1.5	3.27	3.75	3.01	2.25	1.85	44.02	4.99
	2	3.22	3.73	2.94	2.28	1.87	44.29	5.03
T-PSA-NC-5	0.1	3.39	3.82	3.40	2.16	1.82	43.05	4.71
	0.5	3.34	3.79	3.33	2.18	1.83	43.34	4.73
	1	3.29	3.77	3.25	2.21	1.85	43.66	4.75
	1.5	3.25	3.74	3.19	2.23	1.87	43.92	4.77
	2	3.21	3.72	3.11	2.25	1.89	44.17	4.81

**Table 4:** Diameter peaks of 1 wt.% of T-PSA NC and pure TiO_2_ NFs ultrasonically dispersed in EG.

Sample	Diameter Peak 1 (nm)	Diameter Peak 2 (nm)
T-PSA NC1	1158.4	156.03
T-PSA NC2	530	PNS *
T-PSA NC3	399	PNS * PNS *
T-PSA NC4	510.3	PNS *
T-PSA NC5	554.2	PNS *
PSA	241.3	-
TiO_2_	288.7	-

PNS * = Peak is not having significant intensity.

**Table 5:** Zeta potential value and associated suspension stability [123,124].

Zeta Potential (+ or −mV)	Stability
0	Little or no stability
15	Some stability, but settling lightly
30	Moderate stability
45	Good stability; possible settling
60	Very good stability; little settling likely

**Table 6:** Zeta potential value for concentration (vol. 1%) and sonication time for (30 min).

Sample	Zeta Potential Value (mV)	Stability
T-PSA NC1	33.0	Moderate stability
T-PSA NC2	28.0	Moderate stability
T-PSA NC3	38.1	Moderate stability
T-PSA NC4	24.0	Moderate stability
T-PSA NC5	−1.4	Some stability, but settling lightly
PSA	8.1	Some stability, but settling lightly
TiO_2_	−12.7	Some stability, but settling lightly

## Data Availability

Data will be available on request.

## Supplementary Materials

The following supporting information can be downloaded at: https://www.mdpi.com/article/10.3390/nano13162332/s1, Table S1: A brief literature on thermal conductivity and related information for the nanofluids having TiO_2_/PANI/Silica included composites as solid component. References [101,113,125,126,127,128,129,130,131,132,133,134,135,136,137,138,139] are cited in the supplementary materials.
