# Supplementary material for: Studies on the Functional Properties of Titanium Dioxide Nanoparticles Distributed in Silyl–Alkyl Bridged Polyaniline-Based Nanofluids
Source: Nanomaterials (Basel). 2023 Aug 14;13(16):2332. doi: 10.3390/nano13162332 (PMC10459259; doi:10.3390/nano13162332)
Supplement: Supplementary file 1 [file nanomaterials-13-02332-s001.zip › nanomaterials-2477967-supplementary.pdf]

Supporting information

Table S1. A brief literature on thermal conductivity and related information for the nanofluids having TiO2 / PANI/Silica included composites as solid component

Solid component	Base fluid	Thermal conductivity enhancement (%)	Reference
TiO2/Cu	water	2.72	[125]
TiO2/SiO2 composite	Ethylene glycol	1.2	[126]
TiO2 –SiO2 hybrid	Ethylene glycol	22.1	[101]
TiO2-ZnO	Ethylene glycol	32	[127]
CuO/PANI	water	12	[128])
CuO	Ethylene glycol	54	[113]
T-PSA NC1- T-PSA NC5	Ethylene glycol: water	84.9 to 86.7 (Table 1; main paper)	Present work
TiO2	water	32.8	[129]
TiO2	Water*	53.9	[130]
TiO2	Ethylene glycol	12.1	[131]
TiO2	Ethylene glycol: water	46.1	[132]
Silica	water	2.32	[133]
**Silica	water	28.34	[134]
Silica (non porous)	Ethylene glycol	17.4	[134]
Mesoporous silica	Ethylene glycol: water	22	[135]
PANI (un doped)	Water/surfactant	1.20	[136])
PANI (doped)	Water/surfactant	2.35	[137]
PSA	Ethylene glycol: water	84.3 (Table 1; main paper)	Present work

Thermal conductivity values. Water = 0.607 W/m.K; ethylene glycol= 0.254 W/m.K ([136,138,139]; Details like temperature, volumetric/mass (%) of solid component and other details can be referred at the respective references. PANI=Polyaniline, * Corrugated tube, ** sonication effect

Filename: nanomaterials-2477967-supplementary.docx
Directory: E:\2023\8\14\pdf\22
Template: C:\Users\MDPI\AppData\Roaming\Microsoft\Templates\Normal.dotm
Title:
Subject:
Author: 19683
Keywords:
Comments:
Creation Date: 2023/8/13 3:28:00
Change Number: 5
Last Saved On: 2023/8/14 10:12:00
Last Saved By: MDPI
Total Editing Time: 3 Minutes
Last Printed On: 2023/8/14 15:50:00
As of Last Complete Printing
Number of Pages: 1
Number of Words: 191 (approx.)
Number of Characters: 1,092 (approx.)
